# Approximating lattice similarity

**DOI:** 10.1107/S2053273323003200

**Published:** 2023-07-24

**Authors:** Lawrence C. Andrews, Herbert J. Bernstein, Nicholas K. Sauter

**Affiliations:** a Ronin Institute, 9515 NE 137th Street, Kirkland, WA 98034-1820, USA; b Ronin Institute, c/o NSLS-II, Brookhaven National Laboratory, Upton, NY 11973-5000, USA; c Lawrence Berkeley National Laboratory, 1 Cyclotron Road, Berkeley, CA 94720, USA; Universidad del País Vasco, Spain

**Keywords:** lattice matching, Delaunay, Delone, Niggli, Selling

## Abstract

A method is proposed for choosing unit cells for a group of crystals so that they all appear as nearly similar as possible to a selected cell. Related unit cells with varying cell parameters or indexed with different lattice centering can be accommodated.

## Abstract 2

A method is proposed for transforming unit cells for a group of crystals so that they all appear as similar as possible to a selected cell.

## 1. Introduction

A common problem in crystallography is to provide a list of the unit cells of several (or many) crystals so that they can be visually compared, making it easier to identify meaningful clusters of crystals of related morphology. Collections of experimental unit-cell parameters have been created based on similarity of morphology [for example, see Donnay *et al.* (1963 ▸)] and, in recent years, the clustering of unit cells from the myriad of images in serial crystallography has become increasingly important (Keable *et al.*, 2021 ▸). We have created a method to group unit cells to serve these needs and have addressed this problem in the space **S** ^6^ (Andrews *et al.*, 2019*b* ▸).

## 2. Background and notation

### 2.1. The space **S** ^6^

Andrews *et al.* (2019*b* ▸) introduced the space **S** ^6^ as an alternative representation of crystallographic lattices. The space is defined in terms of the ‘Selling scalars’ used in Selling reduction (Selling, 1874 ▸) and by Delaunay (1932 ▸; note that in his later publications, Boris Delaunay used the more accurately transliterated version of his surname, Delone) for the classification of lattices. A point **s** in **S** ^6^ is defined by

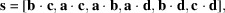

where **d** = −**a** − **b** − **c**. As a mnemonic to remember the order, the terms involve, in order, α, β, γ, **a**, **b**, **c**.

### 2.2. Similarity

In Euclidean geometry, two objects are described as ‘similar’ if they are identical except for a scale factor; see Euclid’s work as translated by Heath (1956 ▸) and a longer description in Wikipedia (https://en.wikipedia.org/w/index.php?title=Similarity_(geometry)&oldid=1097100366). In crystallography, we can say that all face-centered cubic unit cells are similar (assuming that they are in the same presentation). On the other hand, not all primitive orthorhombic unit cells are similar. In a metric space, we refer to two objects as ‘approximately similar’ if the distance between them after scaling to the same size is, in some sense, small, *e.g.* commensurate with the experimental errors in determination of the unit cells. The algorithm below attempts to find the representation of one cell that is nearest to similar to some other cell. For a given reference cell, the probe cell will be transformed to other choices of unit cell that would generate the probe’s lattice and the closest match to the reference will be chosen for the result. Finally, the lattice centering of the reference cell will be restored (if necessary).

## 3. Algorithm

We start with a collection of experimental unit cells. From among them, we select or create the ‘reference’ cell; that is, the one to which all the rest will be matched as closely as possible.

We transform the reference cell by many operations in the course of exploring alternative lattice representations. For each newly generated lattice representation, we accumulate the transformations needed to convert back to the original reference cell. All of these operations are performed in **S** ^6^. (The alternative space, **G** ^6^, is less convenient because the **G** ^6^ fundamental unit is non-convex.) To avoid duplication, for each step we only accumulate transformations that have not already been found.

To begin, each input cell is transformed to the **S** ^6^ representation and then Selling-reduced [see Delaunay (1932 ▸) and Andrews *et al.* (2019*a* ▸), the latter of which discusses the lesser complexity of Selling reduction and includes pseudocode]. As there is a need to be able to reverse the reduction, the reduction transformation is saved for use in later stages.

The following transformations of the reference will be done in three stages.

First, the 24 **S** ^6^ reflections are applied (Andrews *et al.*, 2019*b* ▸) and the results stored. The store of **S** ^6^ vectors and their generating matrices holds 24 entries each at that point.

Because the 24 operations defining reflection are unitary, and in **S** ^6^ they are simply perturbations of the six values, they retain the values and signs of the six values, simply rearranging the six scalars.

Next, the boundary (reduction) transformations (Andrews *et al.*, 2019*b* ▸) are applied to the results of the previous step. The 24 reflections are then applied again. In each step, only newly found results are stored. These last two steps are repeated at least once in order to gain better coverage of possibly useful transformations. The counts of entries for each iteration are 24, 1566, 45 876 and finally 1 016 726. Three iterations, *i.e.* 45 876 entries, have been sufficient in test cases to date.

Although the six scalars are all negative for Selling-reduced unit cells, the boundary transformations are not unitary and so do not retain the six negative values.

Finally, all the accumulated transformed representations of the reference cell must be rescaled and the saved transformations inverted. The **S** ^6^ vectors are all scaled to the same length (see Section 4) and the transformation matrix attached to each vector is inverted, thereby yielding the operation to return a lattice to the vicinity of the original reference cell. For more efficient searching in this final step, it is helpful to use a nearest-neighbor search function such as *NearTree* (Andrews & Bernstein, 2016 ▸).

## 4. Why must the **S** ^6^ vectors be scaled?

**Figure 1:**
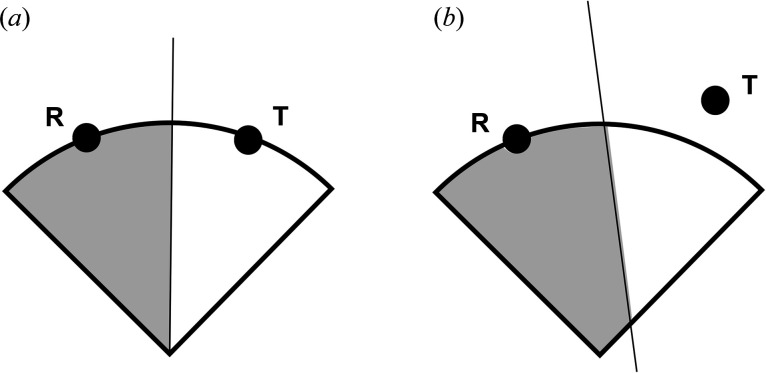
(*a*) The case where the transformed point **T** has been scaled to be at the same distance from the origin as the reference point **R**. (*b*) Point **T** has not been scaled, and some areas are incorrectly assigned to point **R**. In each panel, the straight line between points **R** and **T** separates the regions closer to each of the points.

**Figure 2:**
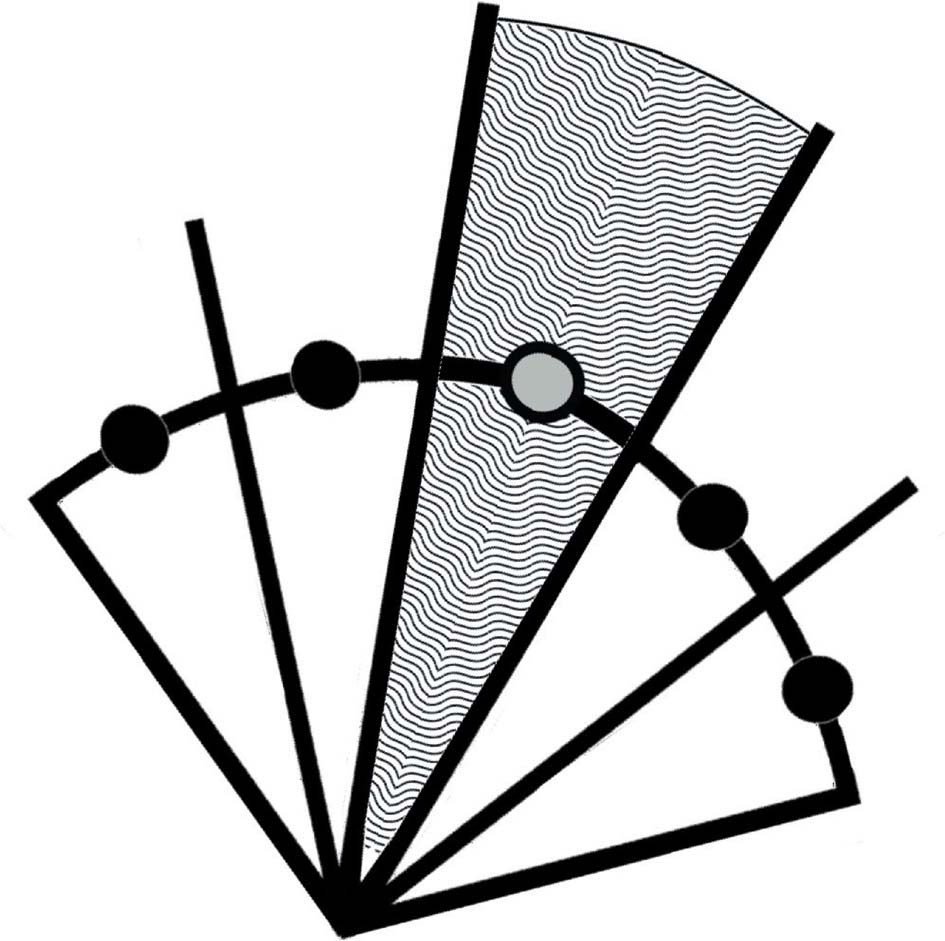
A two-dimensional example of the geometry for determining similarity. Each transformed copy of the reference cell is normalized to a constant length in the chosen space (here **S** ^6^). Each transformed and normalized cell then defines a zone in which every point in that zone is closer to the transformed and normalized cell defining that zone than it is to the transformed and normalized cell defining any other zone. In this example, each point within the textured zone (which extends to infinity) is closer to the gray-centered point than it is to any of the black points.

All similar lattices lie on lines that go through the origin of **S** ^6^. Fig. 1 ▸ shows the distinction between the case where the transformed points are all scaled to be at the same distance from the origin as the reference point [Fig. 1 ▸(*a*)] and the case where they are not [Fig. 1 ▸(*b*)]. Fig. 2 ▸ illustrates the way in which scaling all the reference points to the same 6-spherical surface defines the zones of approximate similarity. Any non-zero scale factor will produce the same correct result. In **S** ^6^ the reflections maintain the distance from the origin but the boundary transformations may not. To repeat: the only way to guarantee that the separation line for two regions goes through the origin is to have all the points at the same radius.

## 5. Angular measure of fit

Because the measure of similarity is independent of scale, projecting points onto a spherical surface does not modify the similarity. The angle between a probe point and the reference point is a meaningful measure of how similar the two points are.

## 6. Generating the approximation

The following operations are performed for each of the probe lattices in the original list. For a given probe lattice, the closest approximation among all of the transformed reference points is found. If there are multiple representations of the reference point that are equally close, then all should be examined. For the case of multiples, a method must be used to find the preferred one. For our purposes, we have found it convenient to choose the one for which the unreduced **G** ^6^ distances to the transformed reference are the smallest. Other choices might be useful for other purposes.

Once the preferred result has been found, the corresponding inverted transformation is used to place the vector in the region of the original reduced reference cell. Finally, the inverse of the reduction operation that was performed on the reference cell is used to create the best match to the original reference. If it is desirable to restore lattice centering, then that operation must also be performed; the search returns a primitive representation of the unit cell.

## 7. Examples

### 7.1. A rhombohedral example

Le Trong & Stenkamp (2007 ▸) cite several structures for phospholipase A2 (krait neurotoxin) that were reported as different structures but were actually all the same structure (Bernstein *et al.*, 2020 ▸). Expanding their search using the program *SAUC* (McGill *et al.*, 2014 ▸), we find a total of six structures, four of which are identical in two pairs. Table 1 ▸ lists the unit cells as reported in the Protein Data Bank (PDB; Bernstein *et al.*, 1977 ▸; Berman *et al.*, 2000 ▸). In Tables 2 ▸, 3 ▸ and 4 ▸, the first entry in each table is used as the reference, and the following five entries are matched as closely as possible to the presentation of the reference cell. In Table 2 ▸, a rhombohedral presentation with PDB ID 1dpy was chosen as the reference. In Table 3 ▸, a *C*-centered cell with PDB ID 1g2x was chosen as the reference. In Table 4 ▸, the hexagonal cell 1u4j was chosen as the reference. In each case, the probe cells were returned in the same presentation, including lattice centering as the reference cell. So the resulting centerings were *hR*, *mC* and *hP*, respectively, for each matched cell, regardless of the input centering, which had been determined by crystallographic analysis.

### 7.2. Adenosine receptor A2A

Unit cells were determined automatically from frames from serial-crystallography data collection for adenosine receptor A2A, PDB ID 5nlx (Weinert *et al.*, 2017 ▸).

Three example unit cells were chosen from several hundred indexed data frames. Two are *C*-centered and one is primitive. Table 5 ▸ gives the reported data, and Tables 6 ▸, 7 ▸ and 8 ▸ are the approximate similarity matches.

### 7.3. Points along a line in **S** ^6^

Tables 9 ▸ and 10 ▸ present two views of artificial data. A line of points in **S** ^6^ was created from the *C*-centered [80.95, 80.95, 57.10, 90, 90.35, 90] to the *A*-centered [57.10, 80.95, 80.95, 90, 90, 90.35] representation of the same cell of phospholipase A2. The series of intervening points interpolated in **S** ^6^ are shown in Table 9 ▸ (each as the reduced unit cell except for the endpoints) and the lattice-matched results are shown in Table 10 ▸.

In Table 10 ▸, the first line is the reference cell, which is also the *C*-centered cell in the first row of Table 9 ▸. The final cell is the same cell but in the *A*-centered presentation. The points between are equally spaced in **S** ^6^ between those two centered points. Table 9 ▸ presents the list of points as generated and Table 10 ▸ lists the same cells in the lattice-matching presentation. Because the initial cell was *C*-centered, the following cells are also in that presentation, although the intermediate cells are not *C*-centered.

### 7.4. Examples from the PDB

The program *SAUC* (McGill *et al.*, 2014 ▸) was used to query the PDB. The search started from the *C*-centered unit cell of PDB entry 1rgx (resistin) requesting the nearest 50 cells; 26 unique cells resulted. Because there was no limit on how far the points could be from the probe, some cells differ significantly from the search cell. The results are listed in Table 11 ▸ in their published representation. Table 12 ▸ lists the same cells in the same order as in Table 11 ▸, but with the same lattice centering as 1rgx, which is the first, reference, entry.

## 8. Summary

A method is proposed for transforming unit cells for a group of crystals so that they all appear as similar as possible to a selected cell. The search for cells similar to the reference cell is done using the reduced cell and comparing with other possible unit cells nearby in the space **S** ^6^. At the end, the lattice centering of the reference cell is restored.

## 9. Availability of code

The C++ code for lattice matching in **S** ^6^ is available on github.com at https://github.com/duck10/LatticeRepLib.git.

## Figures and Tables

**Table 1:** Unit cells of phospholipase A2 from the PDB

PDB ID	Center	*a* (Å)	*b* (Å)	*c* (Å)	α (°)	β (°)	γ (°)
1dpy	*R*	57.98	57.98	57.98	92.02	92.02	92.02
1fe5	*R*	57.98	57.98	57.98	92.02	92.02	92.02
1g0z	*H*	80.36	80.36	99.44	90	90	120
1g2x	*C*	80.95	80.57	57.1	90	90.35	90
1u4j	*H*	80.36	80.36	99.44	90	90	120
2osn	*R*	57.10	57.10	57.10	89.75	89.75	89.75

**Table 2:** The data of Table 1 ▸ matching a rhombohedral reference; the reference cell is highlighted in bold

PDB ID	*a* (Å)	*b* (Å)	*c* (Å)	α (°)	β (°)	γ (°)	Fit (°)
**1dpy**	**57.98**	**57.98**	**57.98**	**92.02**	**92.02**	**92.02**	**0**
1fe5	57.980	57.980	57.980	92.020	92.020	92.020	0
1g0z	57.020	57.020	57.020	90.395	90.395	89.605	2.11
1g2x	57.106	57.106	57.100	89.752	90.248	90.270	2.11
1u4j	57.020	57.020	57.020	90.395	90.395	89.605	2.11
2osn	57.100	57.100	57.100	90.250	90.250	89.750	2.11

**Table 3:** The data of Table 1 ▸ matching a monoclinic reference; the reference cell is highlighted in bold

PDB ID	*a* (Å)	*b* (Å)	*c* (Å)	α (°)	β (°)	γ (°)	Fit (°)
**1g2x**	**80.95**	**80.57**0	**57.10**	**90**	**90.35**	**90**	**0**
1dpy	83.42999	80.53835	57.98115	87.0908	89.9992	90.00114	3.14
1fe5	83.42999	80.53835	57.98115	87.0908	89.9992	90.00114	3.14
1g0z	80.91861	80.35937	57.02143	89.9996	90.5644	90.00144	0.09
1u4j	80.91861	80.35937	57.02143	89.9996	90.5603	90.00144	0.09
2osn	80.92799	80.57842	57.10254	90.0002	90.3502	89.99745	0.01

**Table 4:** The data of Table 1 ▸ matching a hexagonal reference; the reference cell is highlighted in bold

PDB ID	*a* (Å)	*b* (Å)	*c* (Å)	α (°)	β (°)	γ (°)	Fit (°)
**1u4j**	**80.36**	**80.36**	**99.44**	**90**	**90**	**120**	**0**
1dpy	83.4287	80.5380	101.5974	91.6597	90	121.195	4.30
1fe5	83.4287	80.5380	101.5974	91.6597	90	121.195	4.30
1g0z	80.3600	80.3600	99.4400	90	90	120	0
1g2x	80.5809	80.5809	99.3468	90.0138	89.986	120.009	0.09
2osn	80.5752	80.5752	99.3307	90	90	120	0.08

**Table 5:** Adenosine receptor A2A, PDB ID 5nlx, unit cells as reported

Serial No.	Center	*a* (Å)	*b* (Å)	*c* (Å)	α (°)	β (°)	γ (°)
1	*C*	39.741	183.767	140.649	90	90	90
2	*P*	40.160	142.899	92.417	90	102.480	90
3	*C*	180.613	40.156	142.737	90	90.017	90

**Table 6:** Adenosine receptor A2A, approximating a *C*-centered cell The reference cell is highlighted in bold. Centering in parentheses indicates the lattice centering before matching.

Serial No.	Center	*a* (Å)	*b* (Å)	*c* (Å)	α (°)	β (°)	γ (°)	Fit (°)
1	***C***	**39.741**	**183.767**	**140.649**	**90**	**90**	**90**	**0**
2	(*P*)	40.160	180.467	142.899	90	90	89.931	1.37981
3	*C*	40.156	180.613	142.737	89.983	90	90	1.30423

**Table 7:** Adenosine receptor A2A, approximating a primitive cell The reference cell is highlighted in bold. Centering in parentheses indicates the lattice centering before matching.

Serial No.	Center	*a* (Å)	*b* (Å)	*c* (Å)	α (°)	β (°)	γ (°)	Fit (°)
2	***P***	**40.160**	**142.899**	**92.417**	**90**	**102.480**	**90**	**0**
1	(*C*)	39.741	140.649	94.008	90	102.21	90	1.37981
3	(*C*)	40.156	142.73	92.512	89.983	102.535	90	0.08374

**Table 8:** Adenosine receptor A2A, approximating a *C*-centered cell The reference cell is highlighted in bold. Centering in parentheses indicates the lattice centering before matching.

Serial No.	Center	*a* (Å)	*b* (Å)	*c* (Å)	α (°)	β (°)	γ (°)	Fit (°)
3	***C***	**180.613**	**40.156**	**142.737**	**90**	**90.017**	**90**	**0**
1	*C*	183.767	39.741	140.649	90	90	90	1.31
2	(*P*)	180.467	40.160	142.899	90	90	89.930	0.07

**Table 9:** A line of unit cells generated by interpolating between the first and last points in **S** ^6^

Center	*a* (Å)	*b* (Å)	*c* (Å)	α (°)	β (°)	γ (°)
*C*	80.95	80.95	57.1	90	90.35	90
*P*	57.24	57.24	80.68	129.50	94.21	90
*P*	57.24	57.24	80.68	124.46	98.26	90
*P*	57.24	57.24	80.68	119.70	102.36	90
*P*	57.24	57.24	80.68	115.16	106.52	90
*P*	57.24	57.24	80.68	110.78	110.78	90
*P*	57.24	57.24	80.68	106.53	115.16	90
*P*	57.24	57.24	80.68	102.36	119.70	90
*P*	57.24	57.24	80.68	98.26	124.46	90
*P*	57.24	57.2	80.68	94.209	129.50	90
*A*	57.10	80.95	80.95	90	90	90.35

**Table 10:** A list of the same cells in the same order as Table 9 ▸ after transformation to match approximately with the reference cell, which is highlighted in bold

*a* (Å)	*b* (Å)	*c* (Å)	α (°)	β (°)	γ (°)	Fit (°)
**80.950**	**80.950**	**57.100**	**90**	**90.350**	**90**	**0**
80.680	88.685	57.240	86.171	94.210	95.086	5.687
80.680	95.7216	57.240	83.0450	98.260	99.564	12.17
80.680	102.287	57.240	80.280	102.360	103.547	19.35
80.680	108.450	57.240	77.787	106.520	107.167	27.08
80.677	114.284	57.240	75.499	110.775	110.520	35.10
80.680	108.450	57.240	77.780	106.530	107.167	27.09
80.680	102.287	57.240	80.280	102.360	103.547	19.35
80.680	95.722	57.240	83.045	98.260	99.564	12.17
80.680	88.685	57.200	86.172	94.209	95.086	5.70
80.950	80.950	57.100	90	90.350	90	0

**Table 11:** Unit cells from the PDB Cells listed are nearest the *C*-centered cell of 1rgx and keeping only one representative of each protein type. The search was performed using the program *SAUC*.

PDB ID	Center	*a* (Å)	*b* (Å)	*c* (Å)	α (°)	β (°)	γ (°)
1rgx	*C*	49.021	52.475	96.609	90	96.53	90
1r8m	*P*	33.429	95.775	33.665	90	101.67	90
2fxo	*P*	40.157	41.867	97.795	91.11	92.73	107.18
4rne	*C*	37.656	54.197	95.677	90	90	90
3mgd	*C*	57.933	56.341	99.721	90	98.86	90
5yo3	*C*	40.328	50.126	94.237	90	90	90
4gzn	*C*	40.218	60.641	96.119	90	90	90
3vvw	*C*	195.72	37.420	40.280	90	94.66	90
4bhv	*P*	33.078	33.621	99.138	90	96.75	90
3ihu	*C*	54.646	79.135	103.244	90	102.08	90
5wou	*C*	36.429	53.884	94.219	90	90	90
3nhm	*C*	56.616	40.408	99.617	90	102.28	90
5k2l	*P*	29.130	29.130	94.257	90	90	90
3t47	*P*	26.152	94.356	29.196	90	97.19	90
4ruv	*P*	31.376	31.376	94.804	90	90	90
5ed9	*C*	86.371	34.743	99.839	90	101.49	90
1sip	*C*	32.180	62.520	95.760	90	90	90
2sam	*C*	62.700	32.200	96.100	90	90	90
1ytj	*C*	62.300	32.100	96.300	90	90	90
4hhx	*C*	34.790	73.610	95.900	90	90	90
3w92	*P*	31.760	33.552	94.998	90	90	90
167d	*P*	33.200	33.200	96.040	90	90	120
4qeg	*P*	31.237	31.237	93.848	90	90	90
2ygg	*C*	200.700	38.350	34.100	90	91.35	90
1oz7	*P*	37.966	95.258	42.611	90	112.58	90
6nfs	*P*	30.584	34.753	94.679	90	90	90

**Table 12:** Data in Table 11 ▸ best matched to PDB entry 1rgx; the reference cell is highlighted in bold

PDB ID	*a* (Å)	*b* (Å)	*c* (Å)	α (°)	β (°)	γ (°)	Fit (°)
**1rgx**	**49.021**	**52.475**	**96.609**	**90**	**96.53**	**90**	**0**
1r8m	42.374	52.020	95.775	90	90	90.41	2.67
2fxo	48.706	66.020	97.795	89.04	93.21	87.50	3.07
4rne	37.656	54.197	95.677	90	90	90	3.80
3mgd	57.933	56.341	99.721	90	98.86	90	2.91
5yo3	40.328	50.126	94.237	90	90	90	2.84
4gzn	40.218	60.641	96.119	90	90	90	3.86
3vvw	54.979	54.979	99.633	86.02	100.74	85.78	3.90
4bhv	47.165	47.165	99.138	85.27	94.73	89.07	2.71
3ihu	54.646	79.135	103.244	90	102.08	90	5.07
5wou	36.429	53.884	94.219	90	90	90	4.01
3nhm	56.616	40.408	99.617	90	102.28	90	4.39
5k2l	41.196	41.196	94.257	90	90	90	2.65
3t47	41.563	36.677	94.356	90	90	83.65	2.91
4ruv	44.372	44.372	94.804	90	90	90	2.54
5ed9	46.548	67.682	99.839	82.70	100.65	107.73	5.82
1sip	35.158	57.508	95.760	90	90	84.31	4.83
2sam	35.242	57.582	96.10	90	90	84.20	4.83
1ytj	35.042	57.348	96.30	90	90	84.36	4.86
4hhx	40.709	63.858	95.90	90	90	80.10	4.63
3w92	46.200	46.200	94.998	90	90	86.86	2.75
167d	33.20	57.504	96.040	90	90	90	5.26
4qeg	44.176	44.176	93.848	90	90	90	2.54
2ygg	51.318	51.318	102.166	82.83	98.954	96.71	3.40
1oz7	44.886	67.078	95.258	90	90	82.86	4.45
6nfs	46.294	46.294	94.679	90	90	82.70	2.99

## References

[bb1] Andrews, L. C. & Bernstein, H. J. (2016). *J. Appl. Cryst.* **49**, 756–761. 10.1107/S1600576716004039 PMC4886977 27275134

[bb2] Andrews, L. C., Bernstein, H. J. & Sauter, N. K. (2019*a*). *Acta Cryst.* A**75**, 115–120. 10.1107/S2053273318015413 PMC6302928 30575589

[bb3] Andrews, L. C., Bernstein, H. J. & Sauter, N. K. (2019*b*). *Acta Cryst.* A**75**, 593–599. 10.1107/S2053273319002729 PMC6492488 31041913

[bb4] Berman, H. M., Westbrook, J., Feng, Z., Gilliland, G., Bhat, T. N., Weissig, H., Shindyalov, I. N. & Bourne, P. E. (2000). *Nucleic Acids Res.* **28**, 235–242. 10.1093/nar/28.1.235 PMC102472 10592235

[bb5] Bernstein, F. C., Koetzle, T. F., Williams, G. J. B., Meyer, E. F. Jr, Brice, M. D., Rodgers, J. R., Kennard, O., Shimanouchi, T. & Tasumi, M. (1977). *J. Mol. Biol.* **112**, 535–542. 10.1016/s0022-2836(77)80200-3 875032

[bb6] Bernstein, H. J., Andrews, L. C., Diaz, J. A. Jr, Jakoncic, J., Nguyen, T., Sauter, N. K., Soares, A. S., Wei, J. Y., Wlodek, M. R. & Xerri, M. A. (2020). *Struct. Dyn.* **7**, 014302. 10.1063/1.5128498 PMC6952294 31934601

[bb7] Delaunay, B. N. (1932). *Z. Kristallogr.* **84**, 109–149.

[bb8] Donnay, J. D. H., Donnay, G., Cox, E. X., Kennard, O. & King, M. V. (1963). Editors. *Crystal Data: Determinative Tables*, 2nd ed. Buffalo: American Crystallographic Association.

[bb9] Heath, T. L. (1956). Translator. *The Thirteen Books of Euclid’s Elements*, 2nd ed., unabridged. North Chelmsford, Massachusetts, USA: Courier Corporation.

[bb10] Keable, S. M., Kölsch, A., Simon, P. S., Dasgupta, M., Chatterjee, R., Subramanian, S. K., Hussein, R., Ibrahim, M., Kim, I.-S., Bogacz, I., Makita, H., Pham, C. C., Fuller, F. D., Gul, S., Paley, D., Lassalle, L., Sutherlin, K. D., Bhowmick, A., Moriarty, N. W., Young, I. D., Blaschke, J. P., de Lichtenberg, C., Chernev, P., Cheah, M. H., Park, S., Park, G., Kim, J., Lee, S. J., Park, J., Tono, K., Owada, S., Hunter, M. S., Batyuk, A., Oggenfuss, R., Sander, M., Zerdane, S., Ozerov, D., Nass, K., Lemke, H., Mankowsky, R., Brewster, A. S., Messinger, J., Sauter, N. K., Yachandra, V. K., Yano, J., Zouni, A. & Kern, J. (2021). *Sci. Rep.* **11**, 21787.

[bb11] Le Trong, I. & Stenkamp, R. E. (2007). *Acta Cryst.* D**63**, 548–549. 10.1107/S0907444907007354 17372360

[bb12] McGill, K. J., Asadi, M., Karakasheva, M. T., Andrews, L. C. & Bernstein, H. J. (2014). *J. Appl. Cryst.* **47**, 360–364. 10.1107/S1600576713031014 PMC3937814 24587790

[bb13] Selling, E. (1874). *J. Reine Angew. Math.* **1874**(77), 143–229.

[bb14] Weinert, T., Olieric, N., Cheng, R., Brünle, S., James, D., Ozerov, D., Gashi, D., Vera, L., Marsh, M., Jaeger, K., Dworkowski, F., Panepucci, E., Basu, S., Skopintsev, P., Doré, A. S., Geng, T., Cooke, R. M., Liang, M., Prota, A. E., Panneels, V., Nogly, P., Ermler, U., Schertler, G., Hennig, M., Steinmetz, M. O., Wang, M. & Standfuss, J. (2017). *Nat. Commun.* **8**, 542. 10.1038/s41467-017-00630-4 PMC5599499 28912485

